# Multiple sclerosis: time for early treatment with high-efficacy drugs

**DOI:** 10.1007/s00415-023-11969-8

**Published:** 2023-10-18

**Authors:** Krzysztof Selmaj, Bruce A. C. Cree, Michael Barnett, Alan Thompson, Hans-Peter Hartung

**Affiliations:** 1 Department of Neurology, University of Warmia and Mazury, 30 Warszawska Ave, 10-082 Olsztyn, Poland; 2 Center of Neurology, Lodz, Poland; 3 Department of Neurology, Weill Neurosciences Institute, UCSF, San Francisco, USA; 4 Brain and Mind Centre, University of Sydney, Sydney, Australia; 5 Faculty of Brain Sciences, University College, London, London, UK; 6 Department of Neurology, Heinrich-Heine-University, Düsseldorf, Germany; 7 Department of Neurology, Medical University of Vienna, Vienna, Austria; 8 Department of Neurology, Palacky University, Olomouc, Olomouc, Czech Republic

**Keywords:** Multiple sclerosis, High-efficacy drugs, Escalation therapy, Safety

## Abstract

This review addresses current changes in the approach to treating patients with multiple sclerosis (MS). The widely practiced approach of utilizing agents with lower treatment efficacy (LETA) at onset with subsequent escalation has been challenged by new data suggesting that MS patients derive greater benefit when therapy is initiated with high-efficacy treatment agents (HETA). Several recent studies compared treatment efficacy and safety of early administration of HETA versus LETA. The results of randomized, double blind, phase III studies with LETA as a control arm and population-based larger and longer studies using propensity scoring, marginal structural modeling and weighted cumulative exposure analysis support the benefit of early treatment with HETA. Patients initiating their treatment with HETA, regardless of prognostic factors and MRI burden at baseline, showed significantly lower annualized relapse rate (ARR) and reduced disability progression in follow-up periods of up to 10–15 years. Moreover, the safety profile of recently approved HETA ameliorates concerns about off-target effects associated with a number of earlier high-efficacy drugs. Patient perception has also changed with an increasing preference for medication profiles that both improve symptoms and prevent disease progression. Accumulating data from randomized studies and the results of large population-based studies demonstrating short-term and longer-term patient benefits support the view that HETA should be more widely used. The adoption of early treatment with HETA capitalizes on a window of opportunity for anti-inflammatory drugs to maximally impact disease pathology and heralds a sea change in clinical practice toward pro-active management and away from a philosophy routed in generating clinical benefit as a consequence of treatment failure.

## Introduction

The clinical development of drugs to treat relapsing multiple sclerosis (RMS) over the past 25 years created an armamentarium of some 23 disease modifying agents [1]. They are commonly divided into drugs of low or moderate effectiveness (LETA), also termed platform or first-line therapeutics (interferons, glatiramer, teriflunomide, fumarates), and high-efficacy treatment agents (HETA) S1P receptor modulators, natalizumab, anti-CD20 monoclonal antibodies, alemtuzumab and cladribine. However, it should be noted that division between LETA and HETA is not unequivocal and in some studies S1P receptor inhibitors and fumarates are classified in the same group. Two competing strategies have been proposed for MS treatment, namely the escalation or treat-to-target approach initiated with agents of lower treatment efficacy (LETA) which has dominated MS therapeutic paradigms [2] and early, or in some cases first-line, treatment with high-efficacy treatment agents (HETA). The escalation strategy involves initiation of treatment with a drug of low or moderate efficacy, careful monitoring of the treatment response and, if needed, switching to other LETA or HETA. This approach was mainly driven by safety concerns related to mitoxantrone (cardiac toxicity and leukemia) and natalizumab (progressive multifocal leukoencephalopathy). However, with the development of other HETAs with better safety profiles and implementation of risk mitigation strategies to limit side effects the potential to use highly effective therapies for early treatment of RMS patient emerged.

In this paper, we present the evidence to support a shift in the treatment paradigm from escalation to early use of HETA. We reemphasize the need for early treatment initiation regardless of prognostic factors and MRI burden, summarize the safety profiles of LETA and HETA and present results of direct comparison of escalation versus early use of HETA in stringent controlled clinical trials and in real-word population-based studies. It is concluded that early HETA treatment should be considered for the majority of patients diagnosed with MS.

## Case for early treatment

The benefit of early treatment in relapsing MS (RMS) received overwhelming support from numerous studies [3, 4] and is recommended in the current treatment guidelines [5, 6].

Interferon β, glatiramer acetate, teriflunomide and cladribine trials in CIS patients consistently showed a numerically greater reduction in relapse risk (the primary outcome uniformly being time to relapse or definite MS) compared to trials with the same medications in confirmed relapsing MS cohorts [7]. Patients naïve to treatment and patients with shorter disease duration generally showed numerically better clinical and MRI outcomes in double-blind randomized studies, relative to participants with longer disease duration and/or prior treatment experience. In addition, data from open-label extension studies demonstrated that patients with delayed treatment initiation never caught up with those who received the intervention from trial onset. Several sources of real-world data, employing new statistical methods, such as propensity scores, marginal structural modeling and weighted cumulative exposure analysis, also support the benefit of early treatment [8].

The Swedish STOP-MS project involved data from 639 MS patients receiving MS drugs for a median follow-up of 8.25 years [9]. Patients who started treatment 3 years after MS onset reached the irreversible Expanded Disability Status Scale (EDSS) score of 4 sooner with a hazard ratio of 2.64 (1.71–4.08), compared with the patients who started treatment within 1 year from MS onset. These results were recently confirmed in a much larger patient cohort of 11,871 patients with median follow-up time from treatment initiation of 13.2 years, in the first report from the Big Multiple Sclerosis Data (BMSD) network [10]. The risk of reaching the disability outcomes was significantly lower (*p* < 0.0004) for the first quintile patients' group defined as patients treated within 1.2 years of disease onset. In another study from MSBase [11] using marginal structural models, a total of 14,717 patients were studied. Among 1,085 patients with ≥ 15-year follow-up, treated patients were less likely to experience relapses (0.59, 0.50–0.70, *p* = 10^–9^) and worsening of disability (0.81, 0.67–0.99, *p* = 0.043). This study provides evidence that MS therapies improving disability outcomes in relapsing MS over the long term. Importantly, the positive effect of early intervention was observed both for relapse-dependent progression (RDW) as well as in patients with relapses independent progression (PIRA) [12].^.^

The impact of MS treatment on the different types of disability worsening in the randomized placebo-controlled phase 3 trials showed that MS drugs reduced the proportions of patients who had all-cause disability worsening events, with the strongest effect in remitting relapsing MS (RRMS). However, in secondary progressive (SPMS) and primary progressive (PPMS), MS-treated patients also experienced a reduction in 6-month CDW events compared with of placebo-treated patients [13]. These data indicated that early treatment in progressive form of MS also slowed down disability development more efficiently than delaying therapeutic intervention.

## Limitations of prediction markers

In the decision-making process for the initial choice of treatment, predictive factors may have an important role. From natural history studies, factors associated with delaying time from symptomatic onset to cane dependency or SPMS include: younger age of onset, relapsing onset MS versus primary progressive MS, female sex, occurrence of optic neuritis, absence of motor symptoms at onset, absence of cerebellar symptoms at onset, and monosymptomatic onset [14, 15]. Based on these risk factors, patients with a seemingly benign prognosis are often recommended to start with lower-efficacy agents. Although several studies evaluated factors heralding a poor prognosis in MS, the link between prognostic factors and disease course remains poorly understood and MS remains a highly unpredictable disorder. In an early study of the natural history of MS, a series of models was developed to predict disability outcome [14]. Compared to the median time to cane dependency, the models reduced the prediction error by up to 39%. The authors noted that there was substantial unexplained variation and therefore the models were relevant only at the group level. Further, the influence of treatment greatly outweighs the relatively minor effects of baseline predictors. The relationship between early clinical characteristics and long-term disability outcomes in the 16-year cohort follow-up of the pivotal interferon β-1b trial showed that baseline MRI measures of atrophy and lesion number did not correlate with later physical or cognitive outcomes [16]. In two other studies assessing conversion to SPMS, several inconsistencies were found. In one study, neither sex nor symptom type at presentation influenced conversion to SPMS [17], and in another study, polyfocal initial symptoms were not predictive of conversion to SPMS in a study of over 8000 patients [18]. The current understanding of MS disease prediction does not allow for a single measure (s) to be identified. Therefore, the implementation of prognostic factors as prerogatives for treatment choice is challenging and it is not possible to accurately predict prognosis and treatment response at disease onset for an individual patient.

Recently, newer ways to discern patients at higher risk for progression have been investigated. Several phase 3 trials and population-based studies suggest that serum or CSF concentration of serum NfL (neurofilament light) may be a promising predictor of subsequent relapse rate, worsening of disability, MRI lesion activity, and brain volume loss in all forms of MS, including CIS. However, NfL is not specific to MS and a number of confounding factors may limit its utility in the application to individual patients with MS [19]. Similar limitations may apply to the measurement of brain atrophy and its use for the prediction of the further course of MS.

In the decision-making process how to initiate MS treatment, an important role was devoted to benign MS. However, the existence and prevalence of benign MS is controversial. In a recent study evaluating the prevalence of benign multiple sclerosis using a definition that extends the EDSS by including measures of impaired cognition and effects on employment in a population of 1049 patients with disease duration of > 15 years, only nine individuals were identified that had truly benign MS [20]. Similarly, in another recent report based on data exported from the German MS Registry and using more stringent definitions of benign MS, only 13% of patients met the diagnosis [21]. Using data from the ReLSEP, a French population-based registry, 26% of patients maintained the status of “benign MS” (EDSS < 3.0) between 10 and 30 years from onset [22]. Importantly, both the German and French registries included actively treated MS patients in their analyses and therefore the estimates of benign MS in both these studies are likely to be confounded by the impact of treatment on preventing worsening disability. For MS patients with an initial relapsing–remitting course, the probability of non-progressive disease after 40 years was 22% and after 50 years it was 14% [23]. In addition, follow-up studies of patients with MS with disease classified as ‘benign’ after 10 years showed that a large proportion of these patients later converted to a secondary progressive course [24].

## HETA: concept validation from the pathology of MS

One of the most important justifications to initiate HETA early is the irreversible pathology of MS. Specifically, acute axonal loss is maximal in early MS and relates primarily to lesion activity [25]. Demyelinated axons, surviving acute lesion formation, have limited potential for remyelination, particularly with increasing disease duration and age, and are susceptible to ongoing degeneration. Structural axonal changes cannot be repaired and are believed to, at least in part, underlie irreversible disability [25]. Initially, the primary events driving early MS pathology was attributed to focal inflammatory foci visualized on MRI as gadolinium enhancing T1 and new/enlarging T2 lesions. A second wave of pathology dependent on neurodegenerative mechanisms occurs with disease development. Recent studies have challenged this dual mechanism with demonstration of compartmentalized inflammation within the CNS in the progressive forms of MS [26]. The activity of MS lesions in SPMS and PPMS tended to be restricted to chronic active lesions, meningeal lymphocytic infiltrates and, most recently, to microglia activation and smoldering pathology [27]. Most of HETAs suppress focal inflammatory processes within the CNS more efficiently than moderately effective drugs. For example, ocrelizumab and ofatumumab, after 2 years of treatment, reduced gadolinium-enhanced T1 lesions by 95% and 96%, respectively, versus interferon β-1a or teriflunomide [28, 29]. In addition, a number of HETAs showed good penetration to the CNS and express their anti-inflammatory activity in situ. Thus, it is critically important to stop the inflammatory pathologic process as early as possible to prevent accumulation of tissue damage and disease burden in all MS forms.

## Safety—are LETA completely safe?

For the last twenty-five years, it was widely accepted that LETA have a good safety profile. By contrast, a broad perception that HETA carry greater safety risks has limited their uptake. However, closer scrutiny of the safety of injectable drugs suggests that this is not entirely the case. For example, over 30% of patients treated with interferon-β develop increased aminotransferases, with > 10% exhibiting grade 2 and > 3% grade 3 elevations [30]. Up to 35% of patients in clinical trials were reported to have an adverse event involving cytopenia affecting leukocytes, lymphocytes, neutrophils and platelets [31]. In a cohort of 787 of patients with RRMS followed up for 8 years, an increased prevalence of thyroid dysfunction and thyroid autoimmunity (TA, > 10%) was observed in interferon treated patients [32]. Thrombotic microangiopathy, a serious condition, was reported in several patients on long-term treatment with interferon β [33]. Whether patients treated with interferon β have a higher incidence of depression remains contentious. In addition to safety concerns, poor tolerability related to flu-like symptoms occurs in 30–60% of patients during interferon-β treatment and is a frequent cause for treatment withdrawal [34].

Patients treated with glatiramer may experience injection-site reactions, lipoatrophy, rash, skin necrosis, leukopenia and thrombocytopenia, and immediate post-injection systemic reactions [35]. The most common adverse events occurring during teriflunomide treatment include diarrhea, nausea, increased ALT levels, severe hepatotoxicity, bone marrow suppression, opportunistic infections, increased blood pressure, peripheral neuropathy, alopecia, and interstitial lung disease [36]. For dimethyl fumarate (DMF) and monomethyl fumarate (MMF), flushing and gastrointestinal symptoms, including diarrhea, nausea and abdominal pain, are common. DMF treatment may also significantly decrease lymphocyte counts [37]; associated immunosuppression may rarely be associated with opportunistic infections [38].

The magnitude of the increased potential risk of infections was evaluated in pwMS treated with HETA versus injectable therapies in real-world populations. The rate remained significantly higher for rituximab (HR, 1.70 [95% CI, 1.11–2.61]) but not fingolimod (HR, 1.30 [95% CI, 0.84–2.03]) or natalizumab (HR, 1.12 [95% CI, 0.71–1.77]) compared with interferon beta and GA [39].

## Safety—changing perception of HETA

Negative perceptions of the safety of HETA may have arisen from the high frequency of adverse events that occurred during therapy with the first agents, mitoxantrone (cardiomyopathy and promyelocytic leukemia) and natalizumab (progressive multifocal leukoencephalopathy) and then later with alemtuzumab (de novo autoimmune disease and thromboembolic events) [40]. However, the results of recent long-term HETA safety data have reversed this negative opinion considerably. For example, the safety data from Longterms, the longest (14 years) follow-up study of fingolimod showed that annual frequencies of AEs remained low and no new safety findings emerged over the long term [41]. However, it should be noted that the safety profile of HETA varies by individual drugs.

Natalizumab has a high risk for JC virus (JCV) reactivation and PML development, a disease with potential serious neurological consequences and a 25% mortality. The rate of PML in JCV positive patients depends on previous immunosuppressive treatment and length of exposure and can be as high as 7/1000 [42]. The risk stratification of PML involves an index of anti-JCV serum antibodies. Recently, studies with expanded interval dosing provided additional data on mitigation of the PML risk in patients treated with natalizumab [43]. Nevertheless, natalizumab administered to JCV-negative patients is an excellent option to treat MS patients with high disease activity leading to its prompt and effective stabilization. However, these patients should be carefully monitored for possible seroconversion with JCV antibody assessment every 6 months. Another complication of natalizumab treatment is rebound activity following treatment discontinuation which should be effectively overcome by switching to another therapy, e.g., anti-CD20 monoclonal antibodies.

Siponimod, shown to be efficacious in people with SPMS, was the first sphingosine-1-phosphate-receptor (S1PR) modulator for which dose titration was recommended at treatment initiation to minimize bradycardia and the occurrence of AV block [44]. The recently approved selective S1PR1 and S1P5 modulator, ozanimod, studied in relapsing MS also showed a favorable safety profile; and no first dose observation is required in patients without pre-existing cardiac conditions [45]. Hepatotoxicity and macular edema were also significantly less frequent than during treatment with the non-selective S1PR modulator fingolimod.

Cladribine, a synthetic purine nucleoside administered in two annual cycles, is associated with lymphopenia and a slight increase in infection rates**.** Ameliorating initial concerns, a long-term observational follow up program did not detect a higher risk of malignancies in patients treated with this treatment regime [46]. At present, similar data are not yet available for patients that received more than two cycles.

In a nationwide registry-based cohort study from the Swedish MS register and the Swedish Cancer Register, no increased risk of invasive cancer was seen with rituximab and natalizumab, compared to the general population. However, there was a borderline-significant increased risk with fingolimod, compared to both the general population and rituximab [47].

Alemtuzumab generated major concerns about safety among HETA. However, in a long 6 years extension study, infections declined each year following initiation of alemtuzumab treatment, with serious infection incidences ≤ 4.0% in years 3–6 [48]. Alemtuzumab can evoke a number of secondary autoimmune conditions. The most common is thyroid disorders followed by thrombocytopenia, glomerular nephropathies, autoimmune hemolytic anemia, autoimmune hepatitis and hemophagocytic lymphohistiocytosis. Recently, alemtuzumab was associated with cerebrovascular complications, arterial dissections and stroke [49]. Safety of the anti-CD20 monoclonal antibodies ocrelizumab, ofatumumab and ublituximab were evaluated in short- and long-term studies. In the pivotal OPERA phase 3 twin studies of ocrelizumab, the most common adverse event was infusion-related reactions, with very low frequencies (0.3%) of pure allergic events. The frequency of infections was slightly higher in ocrelizumab versus interferon β-1a (58.4% vs. 52.5%, respectively). However, serious infections were less common in ocrelizumab when compared to interferon 1a (1.3% vs. 2.9%) [28]. Additionally, in the ASCLEPOIS twin trials of ofatumumab, upper respiratory tract infections were less common and the number of any adverse events was slightly lower in ofatumumab treated patients than in the teriflunomide arm [29]. In the ULTIMATE studies, ublituximab was associated with more frequent infusion reactions yet infections were similar between the two groups [50]. There have been 10 cases of PML in patients treated with ocrelizumab (post-marketing data on file) [51] and all but one were carry-over cases from a prior DMT (natalizumab and fingolimod). In the long term, infection analysis in pwMS treated with ocrelizumab, up to 8 years, the rate of infection did not increase. No specific tumor risk, including breast cancer, was reported for ocrelizumab in the post-marketing analysis [52]. Anti-CD20 monoclonal antibodies decreased levels of serum immunoglobulins IgA, IgG and predominantly IgM. Most importantly, no correlation was found between lowered serum levels of immunoglobulins and the rate of infections [53]. However, long-term data on hypogammaglobulinemia showed continued decreased level of IgG and IgM in pwMS treated with rituximab and ocrelizumab. The question is how long this trend will be maintained and how much it may affect immune responses. Additional concern about anti-CD20 therapies was raised in relation to severity of Covid infection in patients treated with anti-CD20. The risk of breakthrough SARS-CoV-2 infections was higher in patients treated with ocrelizumab and fingolimod [54]. It was also reported that humoral immune response is seriously impaired and may pose some risk for effective vaccination with SARS-CoV-2 mRNA vaccine in patients treated with ocrelizumab and rituximab [55]. However, anti-SARS-Cov2 T cell responses are not affected by these drugs.

## HETA versus LETA—controlled short-term randomized trials

The widespread approval of multiple drugs for RMS resulted in a situation in which placebo-controlled studies are ethically unacceptable. Thus, several pivotal studies compared the efficacy and safety of HETA with LETA within the stringent conditions of phase III clinical trial programs. In addition, a large number of patients enrolled in these studies were treatment-naïve creating, a unique opportunity to evaluate the short-term efficacy of an escalation strategy with LETA versus early HETA on treatment initiation (Table 1).

**Table 1:** Short-term randomized HETA studies vs. LETA

Study	HETA	Comparator	Duration	Reduction					NEDA
				ARR	3mCDW	Gd+	T2	BV	T2
Transforms [55]	Fingolimod	IM Interferon beta-1a	12 months	52%	ns	59%	30%	27%	63.4% v.44.3%
CARE MS I [56]	Alemtuzumab	SC Interferon beta-1a	24 months	54.9%	ns (27.3%)^a^	51.5%	27%	42%	38.6% v.26.7
CARE MS-II [57]	Alemtuzumab	SC Interferon beta-1a	24 months	49.4%	42%^a^	71%	57.5%	23%	32.2% v.13.6
Opera I [29]	Ocrelizumab	IM Interferon beta-1a	24 months	46%	43%	94%	77%	23.5%	48 v.29%
Opera II [29]	Ocrelizumab	IM Interferon beta-1a	24 months	47%	37%	95%	83%	23.8%	48 v.25%
Radiance [60]	Ozanimod	IM Interferon beta-1a	24 months	38%	ns	53%	42%	27%	24.2% v. 17%
Sunbeam [59]	Ozanimod	IM Interferon beta-1a	12 months	48%	ns	63%	48%	31%	NA
Optimum [61]	Ponesimod	Teriflunomide	24 months	30.5%	ns (17%)	56%^b^		34%	25 v.16.5%
Asclepios I [30]	Ofatumumab	Teriflunomide	24 months	50.5%	34.4^c^	97.5%	82%	ns (7%)	44.6 v. 17.7%
Asclepios II [30]	Ofatumumab	Teriflunomide	24 months	58.5%		93.8%	84.5%	ns (7%)	
Ultimate I [50]	Ublituximab	Teriflunomide	24 months	59.4%	ns	96.7%	92.4%	NA	44.6 v. 15.0%
Ultimate II [50]	Ublituximab	Teriflunomide	24 months	49.1%	ns	96.5%	90%	NA	43.0 v. 11.4%

*ARR* annual relapse rate, *ns* non significant, *3mCDW* 3-month confirmed disability worsening, *BV* brain volume, *ns* not significant

^a^6mCDW

^b^CUALS (combined unique active lesions—Gd+ and new and enlarging T2 lesions)

^c^Pooled data from both studies as per predefined conditions

The first head-to-head HETA versus LETA assessment was the TRANSFORMS study, comparing fingolimod with interferon 1a. ARR was significantly lower (52%) in the fingolimod 0.5 mg group [56]. MRI findings supported the primary results. In both CARE-MS studies, alemtuzumab showed superiority in the reduction of ARR over interferon β-1a. In the CARE-MS I study in treatment-naïve patients, alemtuzumab reduced the ARR by 54.9% versus interferon b-1a [57] and the hazard ratio for sustained accumulation of disability (SAD) was 0.58.

In the OPERA I and II studies, ocrelizumab showed a reduction in ARR versus interferon β-1a of 46% and 47%, respectively [28]. In both studies, ocrelizumab strikingly reduced gadolinium-enhancing lesions compared with interferon β-1a (by 94% and 95%, respectively). Within the pooled OPERA population, > 73% (*n* = 1209) of patients were treatment naïve. In this group ocrelizumab versus interferon b-1a showed an even higher reduction in ARR, 0.16 vs. 0.28 (HR 0.57; 0.45–0.72; *p* < 0.001), and a higher reduction of confirmed 12-week disability worsening, 53 vs. 82 events (HR 0.60; 0.42–0.85; *p* = 0.004).

More recently, two additional anti-CD20 monoclonal antibodies, ofatumumab [29] and ublituximab [50] were evaluated in RRMS. Ofatumumab reduced ARR by 50.5% and 58.5% compared to teriflunomide in two phase 3 studies. In the pooled trial data, the percentage of patients with disability worsening, confirmed at 3 months, was 10.9% with ofatumumab and 15.0% with teriflunomide (HR = 0.66; *p* = 0.002). In ULTIMATE I (*N* = 549) and II (*N* = 545) studies, administration of the glycoengineered anti-CD20 monoclonal antibody ublituximab diminished the ARR by 59.4% (*p* < 0.0001) and 49.1% (*p* = 0.0022) relative to teriflunomide, respectively. Contrast enhancing T1 lesions were lowered by 96.7% and 96.5%, while new/enlarging T2 lesions were reduced by 92.4% and 90.0%, respectively (*p* < 0.0001) [50].

Ozanimod was evaluated in RRMS patients versus interferon β-1a in two phase III studies, SUNBEAM [58] and RADIANCE [59]. In both studies, ozanimod reduced the ARR versus interferon by 48% and 38% (*p* < 0.0001), respectively. In treatment-naïve patients, ozanimod reduced the ARR by 41% (SUNBEAM: HR 0.58; 0·43–0·78, *p* < 0·0001) and 36% (RADIANCE: HR 0·64;0·50–0·82) versus interferon β-1a. Accordingly, gadolinium-enhancing and new and enlarging T2 lesions in the ozanimod arm were significantly lower than in the interferon β-1a arm. In addition, younger patients and those with a shorter MS history also showed a greater reduction in both ARR and active MRI lesions than patients treated with interferon β-1a. Ponesimod, another selective S1PR modulator acting on S1PR1, reduced the ARR versus teriflunomide by 30.5% and combined unique active MRI lesions by 56% [60].

Several small trails suggest that autologous HSCT might be considered as an ultimate HETA for MS treatment [61]. However, the amount of evidence is still not sufficient for broad recommendation of HSCT as early treatment. HSCT may be considered for pwMS who demonstrate substantial breakthrough disease activity despite treatment with HETA or have contraindications to HETA [62]. The long-term efficacy and safety data and the burden of HSCT needs to be resolved in ongoing studies, in particular those comparing HSCT with other HETA drugs, BEAT-MS and STAR-MS.

The major limitation of these findings is their relatively short duration (12–24 months) typical for phase 3 clinical trials. However, it should be emphasized that fewer relapses as well as lower MRI activity in the early stage of MS are the strongest recognized predictors of a subsequent less severe MS course [15]. Thus, fast and efficient suppression of disease activity with HETA within a “window of opportunity” in early, relapsing MS may critically reduce subsequent progression and determine a milder disease course.

## HETA versus LETA—real-world studies

Several observational studies have sought to understand the impact of treatments with varying degrees of efficacy and treatment strategies on long-term outcomes (Table 2). The first study that challenged the presumed benefits of escalation therapy with LETA strategy was a single center study in which neither achieving NEDA during the first two years of the study nor escalation therapy, was associated with improved 10-year disability outcomes (EDSS, T25FW, 9HPTand PASAT) [63]. In a Welsh real-life setting dataset, long-term outcomes were assessed in 592 patients, following early HETA (*n* = 105) (alemtuzumab or natalizumab) versus LETA (*n* = 488) (interferons, glatiramer acetate, dimethyl fumarate, fingolimod, and teriflunomide) [64]. The mean change in EDSS at 5 years for the HETA group was significantly lower than in LETA group (0.3 vs. 1.2). The median time to sustained accumulation of disability was 6 (3.17–9.16) years for HETA and 3.14 (2.77–4.00) years for LETA (*p* = 0.05). The results of this study also found that an escalation approach may be inadequate to prevent unfavorable long-term outcomes. A similar study demonstrated that HETA delayed conversion to the secondary progressive disease stage (HR 0.66) [65]. In addition, when patients treated with interferons or glatiramer acetate were escalated to fingolimod, natalizumab or alemtuzumab within 5 years compared to after 5 years, the risk to transition from relapsing remitting to secondary progressive MS was lower (HR 0.76) suggesting an early window of potential benefit from HETA within 5 years of disease onset.

**Table 2:** Long-term RWD HETA studies v. LETA

Study	HETA	Comparator	Duration	Patients #	Endpoint	Results
Harding [65]	NATZ ALEM	INFb GLA DMF TERF FINGO	5 years	592	EDSS Time to CDW	0.3 v 1.2 3.14 v 6.0 years
Brown [66]	FINGO ALEM NATZ	INFs GLAT	5 years	1555	Conversion to SP	HR 0.66
He [67]	RTX	None	6–10 years	544	EDSS	∆ − 0.98 HR 0.34
	OCR MTX ALEM NATZ				Time to CDW	
Buron [68]	NATZ FINGO ALEM CLAD OCRE	INFs TERF DMF GLAT	4 years	388	EDSS Time to CDW	HR 0.50 HR 0.53
Iaffaldano [69]	FINGO	GLAT	10 years	726	EDSS	∆ − 0.67
	NATZ	INFs				
	MTX	AZT				
	ALEM	TERF				
	OCRE	DMF				
	CLAD					
Simonsen [70]	NATZ	INFs	2 years	694	NEDA	OR 4.6
	FINGO	GLAT				
	ALEM	TERF				
		DMF				
Prosperini [71]	MTX	INF beta 1a	10 years	150	EDSS	4.5 v 5.0
	Cyclophosph	INF beta 1b			Time to CDW	HR 0.48
Spelman [72]	RTX NATZ FINGO	TERF DMF INFs GLAT	3–7 years	4861	EDSS Time to CDW	∆ – 1.3 HR 0.71

*ALEM* alemtuzumab, *AZT* azathioprine, *CLAD* cladribine, *Cyclophosph* cyclophosphamide, *DMF* dimethyl fumarate, *FINGO* fingolimod, *GLAT* glatiramer, *INF* interferon, *MTX* mitoxantrone, *NTZ* natalizumab, *OCRE* ocrelizumab, *RTX* rituximab, *TERF* teriflunomide

In a similar retrospective international observational study from MSBase and the Swedish MS registry [66], treatment results were compared in terms of the time HETA were initiated: 0–2 years (early group, *n* = 213) versus 4–6 years (late group, *n* = 253). Disability outcomes were assessed after 6–10 years (median follow-up time 7·8 years). In the sixth year after disease onset, the mean EDSS score was 2.2 in the early treatment group and 2.9 in the late treatment group. The superiority of early treatment persisted for each year of follow-up until year 10. The adjusted mean difference in EDSS score between groups over the whole follow-up period (6–10 years after disease onset) was – 0.98 points (95% CI – 1.51 to – 0.45; *p* < 0·0001).

A nationwide cohort study from Denmark provides further corroborative evidence for the benefit of early HETA [67]. Patients starting HETA as first-time therapy were compared with a propensity score matched sample of patients starting with LETA (*n* = 194 in each group). HETA was defined according to the EMA classification as natalizumab, fingolimod, alemtuzumab, cladribine, or ocrelizumab. LETA included interferons, teriflunomide, dimethyl fumarate, or glatiramer acetate. At 4 years of follow-up, the probabilities of a 6-month confirmed EDSS score worsening were 16.7% (10.4–23.0%) for patients initiated with HETA and 30.1% (23.1–37.1%) in LETA initiators (HR 0.53, 0.33–0.83, *p* = 0.006). Patients initiating HETA had also a lower probability of a first relapse (HR 0.50, 0.37–0.67). Another important result from this study indicated that a heavy MRI lesion burden did not predict a better response to early institution of HETA.

Recently, supportive data on benefit of early HETA were provided by the Italian MS registry investigators [68]. Disability trajectories were evaluated by applying a longitudinal model for repeated measures of EDSS changes compared with baseline values (delta-EDSS). In total, 363 pairs were included and followed for a median observation time of 8.5 (6.5–11.7) years. The mean delta-EDSS differences between HETA and LETA groups showed a gradual increase from 0.1 (0.01–0.19, *p* = 0.03) at 1 year to 0.30 (0.07–0.53, *p* = 0.009) at 5 years and to 0.67 (0.31–1.03, *p* = 0.0003) at 10 years.

In a cohort of 694 MS patients from a Norwegian population-based registry, the odds ratio (OR) of achieving NEDA on HETA compared to LETA drugs as a first drug in the second year was 4,6 (2,8–7,6; *p* < 0.001) [69]. Patients initiating treatment with HETA with moderate risk for disease activity were more likely to achieve NEDA after 2 years than patients initiating treatment with LETA, 58% versus 37%, respectively. Patients on LETA as first-line therapy also showed poor adherence and were more likely to discontinue treatment than patients on HETA as a first drug (65.2 vs. 29.2%, *p* < 0.001). However, perhaps the most striking result showed that patients on LETA were more likely to discontinue due to side effects than patients on HETA as a first drug (45 vs. 14%, *p* = 0.002).

The benefits of early treatment are also observed with older, aggressive immunosuppressive drugs [70]. In a propensity-matched study fewer patients treated initially with mitoxantrone and cyclophosphamide achieved an endpoint EDSS score greater 6.0 after 10 years of observation in comparison to patients treated with interferons. Unfortunately, in the case of these broad-spectrum immunosuppressive drugs, the side effects were threefold higher.

Recently, an interesting study of 4861 patients investigated national differences in MS treatment strategies for RRMS with disability outcomes [71]. Denmark and Sweden differ (7.6% vs. 34.5%) with the rate of HETA MS treatment initiation. The Swedish treatment strategy was associated with a 29% reduction in the rate of post-baseline 24-week confirmed disability worsening relative to the Danish treatment strategy (hazard ratio, 0.71; 95% CI 0.57–0.90; *p* = 0.004).

## Patient expectation—efficacy and maintenance of QOL

With an increasing number of therapeutics for MS, patient expectations and preferences play important roles in the decision-making process. In a study evaluating RMS patients, patients showed the highest preference for medication profiles that are likely to improve their symptoms and prevent future disability [72]**.** In addition, patients were even willing to accept a level of risk for serious adverse events to derive certain clinical benefits. Specifically, they were willing to accept a much higher risk a 10% or 30% risk of severe adverse events or death, for medications that prevented progression for 25 or 32 years, respectively. In addition, several studies demonstrated patients’ negative preference for frequently injectable drug use in the RRMS population and described the most important DMT attributes as efficacy, mode and frequency of administration, and side-effect profile [72]. The results of the UK study supported the above conclusions and documented the high strength of patient preference for the two efficacy-related attributes (relapse-free rate and no symptom progression) [74].

## Conclusion

Accumulating data from randomized short-term trials and results from population-based studies advocate the more frequent use of HETA on treatment initiation because HETA provides greater benefit to MS patients. However, a definite conclusion about the superiority of HETA over the traditional escalation approach should be generated in a direct comparison of these two strategies in prospective randomized controlled trials. Two such studies are underway, the Traditional versus Early Aggressive Therapy for MS (TREAT-MS) trial (NCT03500328), recruiting 900 participants; and the Determining the Effectiveness of Early Intensive versus Escalation Approaches for the Treatment of Relapsing–remitting MS (DELIVER-MS) trial (NCT03535298), which is enrolling 800 patients. Unfortunately, the results of these two studies will not be known before 2024 and 2026, respectively. Moreover, the primary outcome of DELIVER-MS is brain volume loss rather than sustained disability. In the meantime, one might argue that currently available results provide sufficient evidence to encourage wider early use of HETA for RMS early treatment. The propagation of HETA treatment will allow clinicians and patients to capitalize on a window of opportunity for high-efficacy drugs to exert their maximal anti-inflammatory actions at an early stage of the disease, which is positively correlated with long-term effects. This approach will also enable us to escape from the philosophy of generating clinical benefit at the cost of treatment failure. However, the decision to initiate HETA would require active involvement of the patient, discussion of all potential safety issues and weighing the benefits against the risks for each individual decision. In addition to patients benefits from the change in strategy how to initiate MS treatment, the conclusion on the early use of HETA might also impact the future design of MS trials. New MS drugs might be assessed against control groups of drugs with higher efficacy than LETA. It should be noted that full implementation of the strategy of early use of HETA might be hindered by local administrative regulations. FDA approved many MS drugs as first-line treatment, e.g., S1P modulators, whereas EMA approval maintained some restrictions. We believe that with time and accumulation of new data the access to modern MS medication will be harmonized in different geographical locations. Paradoxically, a HEFT strategy might also have positive pharmaco-economic implications, since it should reduce hospitalization, ambulatory visits, complications associated with treatment switching, disability worsening, and unemployment. Therefore, it seems that we have sufficient evidence and expectation to recommend early HETA treatment for the majority of patients diagnosed with RMS.

## Abbreviations

AAR: Annual relapse rate
AE: Adverse events
CIS: Clinically isolated syndrome
CNS: Central nervous system
DMF: Dimethyl fumarate
EDSS: Expanded Disability Status Scale
HEFT: High-efficacy frontline treatment
HETA: High-efficacy treatment agent
LITE: Lower initial treatment efficacy
MMF: Monomethyl fumarate
mDMT: Moderate disease modifying drug
NEDA: No evidence of disease activity
NfL: Neurofilament light
QOL: Quality of life
PPMS: Primary progressive MS
RMS: Relapsing multiple sclerosis
SAD: Sustained accumulation of disability
SPMS: Secondary progressive MS
S1PR: Sphingosine-1-phosphate-receptor
